# Practical management of patients on apixaban: a consensus guide

**DOI:** 10.1186/1477-9560-11-27

**Published:** 2013-12-31

**Authors:** Christopher Ward, Greg Conner, Geoffrey Donnan, Alexander Gallus, Simon McRae

**Affiliations:** 1 Kolling Institute, University of Sydney; Royal North Shore Hospital, Sydney, NSW, Australia; 2 Cardiovascular Diagnostic Services, Liverpool, NSW, Australia; 3 Florey Institute of Neuroscience and Mental Health; The Austin Hospital, Heidelberg, VIC, Australia; 4 Flinders Medical Center, Bedford Park, SA, Australia; 5 Queen Elizabeth Hospital; Royal Adelaide Hospital, Woodville South, SA, Australia

**Keywords:** Apixaban, Novel oral anticoagulants, Bleeding, Perioperative management

## Abstract

**Background:**

Atrial fibrillation (AF) is a common tachyarrhythmia in Australia, with a prevalence over 10% in older patients. AF is the leading preventable cause of ischaemic stroke, and strokes due to AF have a higher mortality and morbidity. Stroke prevention is therefore a key management strategy for AF patients, in addition to rate and rhythm control. Anticoagulation with warfarin has been an enduring gold standard for stroke prevention in NVAF patients. In Australia, three novel oral anticoagulants (NOACs), apixaban, dabigatran and rivaroxaban are now approved and reimbursed for stroke prevention in patients with non-valvular AF (NVAF). International European Cardiology guidelines now recommend either a NOAC or warfarin for NVAF patients with a CHA_2_DS_2_-VASc score ≥2, unless contraindicated. Apixaban is a direct factor Xa inhibitor with a 12-hour half-life and 25% renal excretion that was found in a large trial of NVAF patients to be superior to warfarin in preventing stroke or systemic embolism. In this trial population, apixaban also resulted in less bleeding and a lower mortality rate than warfarin.

**Methods:**

Clinical experience with apixaban outside of clinical trials has been limited, and there is currently little evidence to guide the management of bleeding or invasive procedures in patients taking apixaban. The relevant currently available animal and *ex vivo* human data were collected, analyzed and summarized.

**Results:**

This multi-disciplinary consensus statement has been written to serve as a guide for healthcare practitioners prescribing apixaban in Australia, with a focus on acute and emergency management.

**Conclusions:**

The predictable pharmacokinetics and minimal drug interactions of apixaban should allow for safe anticoagulation in the majority of patients, including temporary interruption for elective procedures. In the absence of published data, patients actively bleeding on apixaban should receive standard supportive treatment. Quantitative assays of apixaban level such as chromogenic anti-Xa assays are becoming available but their utility is unproven in this setting. Specific antidotes for novel anticoagulants, including apixaban, are in clinical development.

## Background

In Australia, three novel oral anticoagulants (NOACs) have been approved for the prevention of stroke in patients with non-valvular atrial fibrillation (NVAF) and one or more additional risk factors for stroke (prior stroke, prior transient ischaemic attack, prior systemic embolism, age ≥75 [or age ≥65 years associated with one of the following: diabetes mellitus, coronary artery disease, or hypertension], arterial hypertension requiring treatment, diabetes mellitus, heart failure ≥ New York Heart Association Class 2, decreased left ventricular ejection fraction or documented peripheral arterial disease). These are dabigatran, a direct thrombin inhibitor, and two direct factor Xa inhibitors, apixaban and rivaroxaban. All three are also approved in Australia for the prevention of venous thromboembolic events (VTE) in adult patients who have undergone major orthopaedic surgery of the lower limb. Rivaroxaban is approved for the treatment of deep vein thrombosis (DVT) and pulmonary embolism (PE) and the prevention of recurrent venous thromboembolism. These novel oral anticoagulants have short half-lives (dabigatran 12-17 hrs, apixaban ~12 hrs, rivaroxaban 5-13 hrs), predictable pharmacokinetics and few drug-drug and drug-food interactions, compared to warfarin. In addition to their favourable pharmacokinetic profiles, dabigatran 110 mg and rivaroxaban have demonstrated similar rates of stroke and systemic embolism reduction to warfarin in NVAF patients, with dabigatran 150 mg and apixaban demonstrating a superior reduction in stroke and systemic embolism, compared to warfarin [1-3]. Additionally, dabigatran 110 mg and apixaban resulted in significantly less major bleeding, compared to warfarin [1,3]. Although specific antidotes for these agents are currently in development, the lack of a reversal strategy has raised concern among healthcare providers.

## Methods

In the absence of robust clinical data for emergency and peri-operative management of patients receiving apixaban, an expert panel of Australian clinicians from the fields of cardiology, neurology and haematology convened to develop this practical consensus guide for apixaban management in Australia, utilising the currently available animal and *ex vivo* human data.

## Results and discussion

### About apixaban

Apixaban is a direct FXa inhibitor with rapid onset of action, a 12-hour half-life and only ~25% renal excretion [4,5]. Apixaban is indicated in Australia for the prevention of venous thromboembolic events (VTE) in adult patients who have undergone elective total hip or total knee replacement surgery and for the prevention of stroke and systemic embolism in patients with non-valvular atrial fibrillation and at least one additional risk factor for stroke. The recommended dose of apixaban for VTE prophylaxis is 2.5 mg BID. The recommended dose of apixaban for stroke prevention in non-valvular atrial fibrillation (NVAF) is 5 mg BID (2.5 mg BID if ≥2 of the following; ≤60 kg, ≥80 years, serum creatinine level ≥133 μm/L) [5].

The risk of stroke and bleeding must be assessed for each patient before commencing any anticoagulation therapy, including apixaban. Some of the patients excluded from the trials [3,6] had baseline characteristics that were associated with increased risk of bleeding (e.g. recent major bleeding, renal insufficiency [CrCl <25 ml/min], severe hepatic impairment, platelet count <100), and there are no or insufficient data on the use of apixaban in such patients. The clinical trials excluded aspirin doses >165 mg/day or dual anti-platelet therapy [3,6]. The concomitant use of apixaban with anti-platelet agents increases the risk of bleeding [7]. Apixaban should be used with caution when co-administered with NSAIDs (including acetylsalicylic acid) because these medicinal products typically increase the bleeding risk. A significant increase in bleeding risk was reported with the triple combination of apixaban, acetylsalicylic acid and clopidogrel in a clinical study in patients with acute coronary syndrome [8].

### Laboratory measurement of apixaban

At present, there is no validated coagulation assay to measure apixaban effect. As a result of FXa inhibition, apixaban prolongs standard clotting tests such as prothrombin time (PT), activated partial thromboplastin time (aPTT) but with variability between reagents [9]. Increases in clotting times are small at best, and the PT may remain normal (ratio <1.2) at a therapeutic concentration of apixaban [9]. Therefore the PT and APTT are not recommended to assess the pharmacodynamic effects of apixaban [5].

Specialised clotting assays can be used to measure apixaban effects. Anti-FXa activity exhibits a close direct linear relationship with apixaban plasma concentration, reaching maximum values at the time of apixaban peak plasma concentrations. The relationship between apixaban plasma concentration and anti-FXa activity is linear over a wide dose range of apixaban [10]. Although treatment with apixaban at the recommended dose does not require routine laboratory monitoring, measurement of drug level by a chromogenic anti-FXa assay may be useful in exceptional situations where knowledge of the apixaban level may help to inform clinical decisions, e.g. overdose or emergency surgery [5]. Anti-Xa assays are generally available in large Australian teaching hospitals, but may not be routinely performed in smaller institutions or after hours. These assays may also be difficult to access in other countries, with significant delays in reporting. Diagnostic laboratories will need validated, commercial apixaban controls and calibrators to adapt their anti-Xa assays for apixaban. Although commercial research-use only apixaban-specific calibrators are currently available in Australia, a standard curve constructed with commercial LMWH standards was reported to show an equally strong correlation with apixaban plasma concentration (r^2^ = 0.89) as one constructed with apixaban (r^2^ = 0.88) [11]. A dilute prothrombin time (dPT), achieved by diluting the thromboplastin reagent in 100 mmol/L CaCl_2_, has been proposed as an improved assay for factor Xa inhibitors [12]. This modification prolonged the PT measurements at therapeutic concentrations of apixaban and showed greater sensitivity than a standard PT [12]. However, others have found that dPT was no better than PT in terms of sensitivity [9], suggesting that further development of these assays is needed.

The delay between the last intake of the drug and the blood sampling should be considered when assessing apixaban levels, since assays are influenced proportionally to apixaban concentration [9].

### Interactions with other medications

Apixaban is metabolized mainly by CYP3A4/5 and is a substrate of efflux transport proteins P-glycoprotein (P-gp) and Breast Cancer Resistance Protein [5]. Therefore, apixaban is contraindicated in patients who are receiving concomitant treatment with strong inhibitors of both CYP3A4 and P-gp, such as azole-antimycotics or HIV protease inhibitors [5] e.g. ketoconazole, itraconazole, voriconazole, posaconazole or ritonavir.

No dose adjustment for apixaban is required when this is co-administered with less potent inhibitors of CYP3A4 and/or P-gp [5] such as diltiazem, naproxen, amiodarone, verapamil, clarithromycin or quinidine.

The concomitant use of apixaban with strong CYP3A4 and P-gp inducers may lead to reduced apixaban plasma concentrations. No dose adjustment for apixaban is required during concomitant therapy with such agents, however strong inducers of both CYP3A4 and P-gp should be co-administered with caution [5] e.g. rifampicin, phenytoin, carbamazepine, phenobarbital and St John’s Wort.

Famotidine, a typical gastric acid suppressant, does not affect the pharmacokinetics of apixaban [13]. As such, increases in gastric pH due to other gastric acid modifiers (such as other H2-receptor antagonists, proton pump inhibitors, and antacids) or the presence of abnormally elevated gastric pH (e.g. achlorhydria) are unlikely to affect the pharmacokinetics of apixaban [13].

### Starting apixaban

Prior to initiating apixaban, liver function and renal function testing should be performed. The European Society of Haematology 2012 guidelines recommend assessment of renal function (by calculated CrCl) be mandatory for all NOACs, with renal function being assessed annually in patients with normal (CrCl ≥80 mL/min) or mild (CrCl 50–79 mL/min) renal impairment, and 2–3 times per year in patients with moderate (i.e. creatinine clearance 30–49 mL/min) renal impairment [14].

Patients with impaired renal function (≤80 mL/min) were at higher risk for all cardiovascular events during the ARISTOTLE trial, and the incidence of major bleeding increased significantly with increasing renal dysfunction [15]. Apixaban was associated with less major bleeding compared with warfarin for three methods of glomerular filtration rate estimation (Cockcroft–Gault, Chronic Kidney Disease Epidemiology Collaboration and cystatin C) and stroke or systemic embolism occurred less frequently in patients assigned to apixaban than warfarin, regardless of renal function [15].

#### *Switching from warfarin to apixaban*

When switching anticoagulation from warfarin to apixaban, it is important to avoid using both drugs at therapeutic doses simultaneously; it is recommended that the INR is monitored daily after the cessation of warfarin, and that apixaban is not started until the INR is <2.0, typically approximately three days after cessation of therapeutic warfarin [3].

#### *Switching from low molecular weight heparin (LMWH) to apixaban*

As both agents have a similar rapid onset of FXa inhibition and effective half-life, switching anticoagulation from LMWH (e.g. enoxaparin) to apixaban, (and vice versa), can simply be done at the time of the next scheduled dose [5].

### *Switching from apixaban*

An increased risk of stroke was observed during the transition from apixaban to warfarin in clinical trials in patients with non-valvular atrial fibrillation [16]. Discontinuation of apixaban prior to the onset of an effective antithrombotic effect of VKA could result in an increased risk of thrombosis. If anticoagulation with apixaban must be discontinued for any reason other than pathological bleeding, consider coverage with another anticoagulant.

#### *Apixaban to warfarin*

When converting from apixaban to warfarin, continue apixaban for 48 hours after the first dose of warfarin. After 2 days of co-administration of apixaban with warfarin, obtain an INR prior to the next scheduled dose of apixaban. Continue co-administration of apixaban and warfarin until the INR is ≥ 2.0.

#### *Apixaban to low molecular weight heparin (LMWH)*

As both agents have a similar rapid onset of FXa inhibition and effective half-life, switching anticoagulation from apixaban to LMWH (e.g. enoxaparin) and vice versa, can simply be done at the time of the next scheduled dose [5].

### Bleeding management in patients receiving apixaban

In the ARISTOTLE study of apixaban in patients with atrial fibrillation, annual major bleeding events for apixaban compared to warfarin were 2.13% per year versus 3.09% per year (p < 0.001) [3]. Intracranial haemorrhage events were 0.33% per year for apixaban, compared to 0.80% per year for warfarin (p < 0.001) [3].

**Figure 1:**
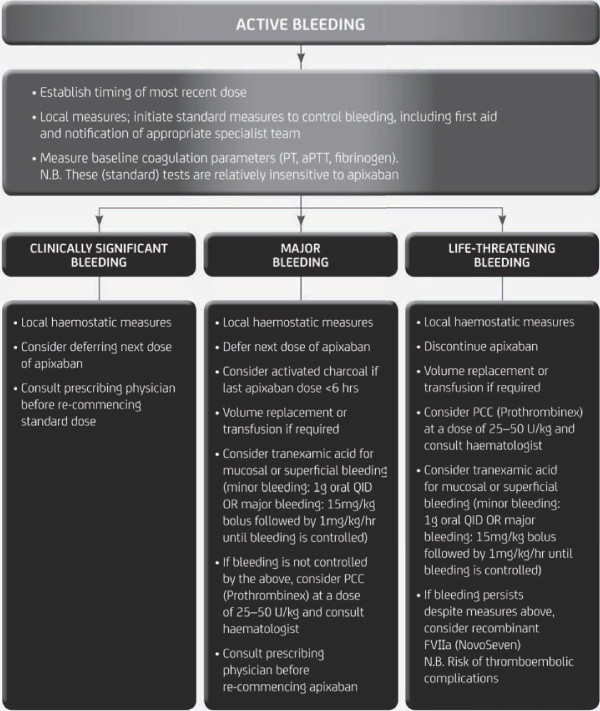
Considerations for the management of bleeding, based on expert consensus.

Spontaneous bleeding may occur with any anticoagulant. In the absence of published data regarding the treatment of patients with active bleeding while receiving apixaban, the following advice for general management of bleeding events is based on expert consensus (Figure 1).

• Establish the primary source of bleeding wherever possible, and secure haemostasis with local measures.

• Most cases of minor bleeding will resolve after cessation of drug, standard supportive treatment, including transfusion, mechanical compression and other local measures.

• If bleeding occurs within 6 hours of last apixaban dose, activated charcoal may reduce apixaban absorption, and hence anticoagulant effect [17]. This should also be considered soon after overdose or accidental ingestion.

• A specific antidote for apixaban is not currently available [5]. Two synthetic molecules are currently in early clinical trials for apixaban reversal. Andexanet alpha (PRT064445) is a truncated form of enzymatically inactive factor Xa, which can dose-dependently reverse the inhibitory activity and correct the prolongation of *ex vivo* clotting times by apixaban and other factor Xa inhibitors [18]. Another synthetic small molecule, aripazine (PER977), appears to have broad activity against the NOACs, reversing the anticoagulant activity of dabigatran, rivaroxaban, apixaban and edoxaban in rat bleeding models [19].

• Apixaban is highly (~87%) protein bound, and hence not expected to be dialyzable [5]. Based on studies of other factor Xa inhibitors in healthy volunteers, prothrombin complex concentrates (PCC) may reverse the anticoagulant effect, however the effect of PCC on clinical bleeding is not proven [20]. When apixaban (200 ng/ml) was added *in vitro* to blood from healthy donors, PCC and activated PCC were more effective at improving thrombin generation than recombinant FVIIa (rFVIIa) [21].

• There is no clinical evidence examining the use of rFVIIa or bypassing agents (FEIBA) in bleeding patients receiving apixaban. In a rabbit model of apixaban-induced bleeding, neither rFVIIa nor PCC reduced blood loss from a standardised hepatosplenic injury, although rFVIIa did reverse prolongation of the prothrombin time and shortened skin bleeding time [22]. When apixaban (200 ng/ml) was added *in vitro* to blood from healthy donors, rFVIIa was more effective than PCC in restoring clotting times and thromboelastography parameters [21]. In animal, *in vitro* and healthy volunteer studies, these agents have partially reversed the anticoagulant effect of apixaban and other factor Xa inhibitors [23-26]. These agents can be considered for life-threatening bleeding, but carry a proven risk of thrombosis.

• There is no evidence to support the use of FFP, other than for volume replacement in case of major bleeding.

### Peri-operative management in patients receiving apixaban

In stable patients, apixaban has a predictable half-life of 8-12 hours, which leaves residual activity of up to 50% at 12 hours and less than 25% at 24-hours after drug cessation [4]. This means that apixaban can be ceased for a shorter period of time than warfarin before invasive procedures, without the routine need to bridge with alternative anticoagulants such as heparin.

Planning for elective surgery or invasive procedures should involve balancing the intervention-associated bleeding risk and thrombotic risk associated with anticoagulant interruption in each individual. A “safe” residual drug level of apixaban for surgery is presently unknown, and no test has been correlated with bleeding risk. As such, there is currently no known threshold at which apixaban patients’ bleeding risk are able to be comparable to non-apixaban treated patients [27].

**Figure 2:**
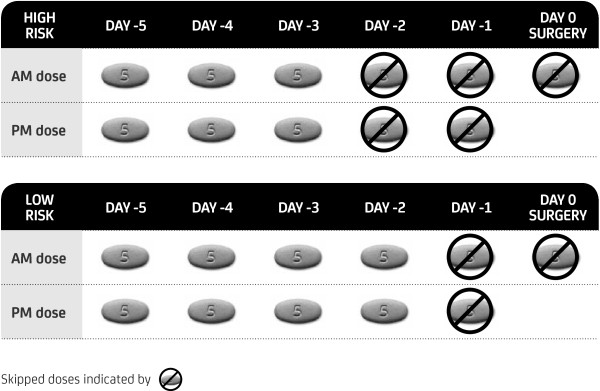
Perioperative dosing/elimination.

In general, apixaban should be discontinued 2 to 3 days prior to elective surgery or invasive procedures [5], as outlined below and in Figure 2. There are small groups of people at higher risk of thrombosis (e.g. CHADS_2_ > 5, recent TIA or stroke) where an individualised approach is needed to minimise the period of sub-therapeutic anticoagulation. A recent review of periprocedural use of antithrombotic therapy notes the importance of checking creatinine clearance in patients on rivaroxaban and apixaban, prior to cessation for high-risk procedures [28]. A longer period of pre-operative discontinuation, up to 5 days, can be considered for patients with renal or hepatic impairment or other conditions associated with decreased drug elimination. In this setting, “bridging” with LMWH has been proposed for patients with a high risk of thrombosis [27]. Given the predictable pharmacokinetics of apixaban, bridging with an alternative anticoagulant should not be required in the majority of cases.

### Advice for assessing peri-procedural dosing

#### *High bleeding risk*

Procedures with a high risk of bleeding (e.g. neurosurgical, urological procedures, major abdominal or orthopaedic): aim to achieve *no* residual apixaban effect at the time of the procedure; last dose of drug should be 3 days prior (5 missed doses including morning of surgery – Figure 2) [29].

#### *Low bleeding risk*

Procedures with a low risk of bleeding (e.g. inguinal hernia repair, percutaneous biopsy, dental extractions): aim to achieve *minimal-mild* residual apixaban effect at the time of the procedure; last dose of drug should be 2 days prior (3 missed doses including morning of surgery – Figure 2) [29].

#### *Minimal bleeding risk*

For selected procedures with minimal risk of bleeding (e.g. cataract surgery, skin cancer excision): therapeutic anticoagulation may be continued.

### Re-commencing apixaban after surgery

Re-commence apixaban dosing only once surgical haemostasis has been secured (typically 24 hours after surgery) [29]. In general, caution should be exercised with re-instituting therapeutic anticoagulation within the first 48 hours after surgery. Where there is a risk of post-operative venous thrombosis and the bleeding risk is high, consider a reduced dose of 2.5 mg BID (recommended prophylactic dose) for the immediate post-operative period.

In patients with poor oral absorption or nil by mouth after surgery, parenteral anticoagulants may be needed until reliable oral absorption is established.

### Neuraxial anaesthesia

Indwelling epidural or intrathecal catheters must be removed at least 5 hours prior to the first dose of apixaban. Experience with neuraxial blockade is limited and extreme caution is therefore recommended when using apixaban in this setting (see P.I.) [5].

## Conclusions

• Apixaban is a direct FXa inhibitor indicated in Australia for the prevention of venous thromboembolic events (VTE) in adult patients who have undergone elective total hip or total knee replacement surgery (2.5 mg BID) and for the prevention of stroke and systemic embolism in patients with non-valvular atrial fibrillation and at least one additional risk factor for stroke (5 mg BID or 2.5 mg BID if ≥2 of the following; ≤60 kg, ≥80 years, serum creatinine level ≥133 um/L) [5].

• In the ARISTOTLE study of apixaban in patients with atrial fibrillation, annual major bleeding events for apixaban compared to warfarin were 2.13% per year versus 3.09% per year (p < 0.001). Intracranial haemorrhage events were 0.33% per year for apixaban, compared to 0.80% per year for warfarin (p < 0.001) [3].

• There is no standardised assay currently commercially available in Australia to measure apixaban effect. As apixaban minimally prolongs PT or aPTT, these clotting tests are not recommended to assess the pharmacodynamic effects of apixaban [5]. A chromogenic anti Xa assay or dilute PT assay may be useful, where knowledge of apixaban exposure is required [10-12].

• Apixaban is contraindicated in patients who are receiving concomitant treatment with strong inhibitors of both CYP3A4 and P-gp, however no dose adjustment for apixaban is required when co-administered with less potent inhibitors. No dose adjustment for apixaban is required during concomitant therapy with strong CYP3A4 and P-gp inducers, however they may lead to reduced apixaban plasma concentrations [5].

• A specific antidote for apixaban is not currently available, however specific anti-Xa inhibitor and universal novel oral anticoagulant antidotes are in clinical development [18,19].

• In the absence of published data regarding the treatment of patients with active bleeding while receiving apixaban, discontinue apixaban, apply standard supportive treatment and other local measures [5]. Activated charcoal may reduce apixaban absorption within 6 h of last dose [17].

• Apixaban is not expected to be dialyzable, however prothrombin complex concentrates (PCC) may reverse the anticoagulant effect and recombinant FVIIa or bypassing agents (FEIBA) can be considered for life-threatening bleeding. FFP will not reverse apixaban effect but can be used as volume replacement in case of major bleeding.

• Planning for elective surgery or invasive procedures should involve balancing the intervention-associated bleeding risk and thrombotic risk associated with anticoagulant interruption in each individual.

## Abbreviations

APTT: Activated partial thromboplastin time; ASA: Acetylsalicylic acid; CrCl: Creatinine clearance; DVT: Deep vein thrombosis; FEIBA: Factor VIII inhibitor bypassing agent; FX: Factor X; INR: International normalized ratio; NOAC: Novel oral anticoagulants; NSAID: Non-steroidal antiflammatory drugs; NVAF: Non-valvular atrial fibrillation; PCC: Prothrombin complex concentrates; PE: Pulmonary embolus; PT: Prothrombin time; rFVIIa: Recombinant activated Factor VII; VTE: Venous thromboembolism.

## Competing interests

The authors received honoraria and travel support from Pfizer Australia to review the literature and attend a working group meeting where this consensus statement was generated.

## Authors’ contributions

CW contributed to consensus statement and wrote the manuscript; GD contributed to consensus statement and reviewed the manuscript; ASG contributed to consensus statement and reviewed the manuscript; GC contributed to consensus statement and reviewed the manuscript; SM contributed to consensus statement and reviewed the manuscript. All authors read and approved the final manuscript.
